# Stereotactic lesioning of cholinergic cells by injection of ME20.4 Saporin in the nucleus basalis of Meynert in a rhesus monkey (*Macaca mulatta*)

**DOI:** 10.1093/jnen/nlaf081

**Published:** 2025-07-17

**Authors:** Muhammad Nazmuddin, Marieke A Stammes, P Christiaan Klink, Marit K Vernes, Jaco Bakker, Jan A M Langermans, Teus van Laar, Ingrid H C H M Philippens

**Affiliations:** Department of Neurology, University of Groningen, University Medical Center Groningen, Groningen, The Netherlands; Department of Physiology and Medical Biochemistry, Faculty of Medicine, Airlangga University, Surabaya, Indonesia; Faculty of Health, Medicine and Natural Sciences (FIKKIA), Airlangga University, Banyuwangi, Indonesia; Animal Science Department, Biomedical Primate Research Centre (BPRC), Rijswijk, The Netherlands; Department of Vision & Cognition, Netherlands Institute for Neuroscience, Royal Netherlands Academy of Arts & Sciences, Amsterdam, The Netherlands; Laboratory of Visual Brain Therapy, Sorbonne Université, Institut National de la Santé et de la Recherche Médicale, Centre National de la Recherche Scientifique, Institut de la Vision, Paris, France; Animal Science Department, Biomedical Primate Research Centre (BPRC), Rijswijk, The Netherlands; Animal Science Department, Biomedical Primate Research Centre (BPRC), Rijswijk, The Netherlands; Animal Science Department, Biomedical Primate Research Centre (BPRC), Rijswijk, The Netherlands; Department Population Health Sciences, Animals in Science & Society, Faculty of Veterinary Medicine, Utrecht University, Utrecht, The Netherlands; Department of Neurology, University of Groningen, University Medical Center Groningen, Groningen, The Netherlands; Animal Science Department, Biomedical Primate Research Centre (BPRC), Rijswijk, The Netherlands

**Keywords:** cholinergic depletion, FEOBV-PET, non-human primate, nucleus basalis of Meynert, Saporin, stereotactic injection

## Abstract

Cholinergic degeneration in the nucleus basalis of Meynert (NBM) is clinically linked to cognitive impairment and gait dysfunction in Alzheimer’s disease and Parkinson’s disease. Modeling cholinergic degeneration in an animal model may provide powerful opportunities to study the clinical-physiological role of the NBM and lead to new therapies. We describe a procedure to inject ME20.4 Saporin, an immunotoxin that specifically binds to and depletes cholinergic neurons stereotactically into the NBM of a rhesus monkey (*Macaca mulatta*). A digital non-human primate brain atlas was co-registered to the brain of the monkey. A custom-designed cranial chamber was also implanted to the skull to guide the injection. The effects of the ME20.4 Saporin injections were evaluated in vivo with PET-CT using [^18^F]-FEOBV as a radiotracer. This approach yielded reliable spatial accuracy and successful delivery of ME20.4 Saporin into the NBM. [^18^F]-FEOBV PET analyses revealed reduced radiotracer uptake in the NBM. Postmortem assessment showed a reduction of ME20.4-positive cells within the NBM. No clear effects on cognitive testing were observed. This Saporin-mediated selective destruction of cholinergic neurons in the NBM, using MRI-guidance and a cranial chamber, offers a promising method to study the pathophysiology of NBM degeneration and possible therapeutic interventions.

## INTRODUCTION

The nucleus basalis of Meynert (NBM) is a non-encapsulated and borderless region in the basal forebrain that consists predominantly of magnocellular cholinergic cells. The NBM is the basis of a widespread cholinergic innervation throughout the cortex and the limbic system.^1^ Current evidence points to the significant physiological and behavioral role of the NBM in cognitive processes including arousal, attention, memory formation, and movement control.^2–4^ Neuronal loss in the NBM is already present in the prodromal stages of Alzheimer disease (AD) and Parkinson disease (PD), prior to cognitive decline and motor symptoms.^5–7^

Preclinical rodent studies have also deciphered the physiological, behavioral, and therapeutic significance of the NBM. However, their clinical translational validity, especially in the context of therapeutic targets, is limited by the significant differences between rodent and human brains.^8^ Attempts to enhance NBM cholinergic activity in patients with AD and Lewy body dementia based on results obtained in rodents have yielded suboptimal results in clinical trials, leaving room for improvement.^9–11^ In contrast to rodents, rhesus monkeys (*Macaca mulatta*) more closely resemble the compartmentalized NBM structure found in humans. Therefore, rhesus monkeys are more suitable to reveal the physiological-behavioral roles of the specific compartments of the NBM.^12–14^ This will serve the development of future therapeutic strategies of the NBM.

A stereotactic neurosurgical technique is required to manipulate the NBM, either by local injection of a pharmacological agent or electrode stimulation.^15^^,^^16^ Early stereotactic approaches in rhesus monkeys relied upon standard 2D brain atlases and a rigid stereotactic head frame to determine and navigate to standardized target coordinates. However, due to the small size of the NBM and considerable anatomical variability across individuals,^17–19^ a personalized approach is needed. Therefore, computed tomography (CT) and/or magnetic resonance imaging (MRI), acquired before and during the stereotactic procedures, to aid intracerebral navigation, is necessary.^16^^,^^20–26^

This paper describes a method of depleting cholinergic projection neurons of the NBM of a rhesus monkey by injecting the cholinergic toxin ME20.4 Saporin bilaterally into the NBM in 2 stages, with the aim of developing an animal model of NBM functioning, closely resembling the human situation.

## METHODS

This study was performed under project license AVD5020020186345 which was issued by the competent national authorities (Central Committee for Animal Experiments) according to Dutch law, article 10a of the “*Wet op de Dierproeven*.” Approval was obtained by the institutional animal welfare body from the Biomedical Primate Research Centre (BPRC; Rijswijk, The Netherlands). All procedures, husbandry, and housing were performed in accordance with the Dutch laws on animal experimentation and the EU Directive 63/2010. The BPRC is accredited by *AAALAC International*.

### Animal, housing, and husbandry

One male rhesus monkey (*Macaca mulatta*) (7 years, 11.7 kg), which was born and bred at the BPRC, was studied. The animal was determined to be healthy according to physical examination and the evaluation of routine hematology and serum chemistry before inclusion in this study. During the study, the animal was socially housed with another male rhesus monkey. The enclosure measured 2 m in height with a surface area of 4 m^2^ with wood fiber bedding. The room temperature was 20-24 ± 2° C, with 6 air changes per hour, a 12 h light–dark cycle, and relative humidity of 50 ± 10%. The animal was offered a daily diet consisting of commercial monkey pellets (ssniff, Soest, Germany), supplemented with limited amounts of vegetables and fruit. Homemade and commercially available enrichment products were provided daily. Drinking water was available ad libitum via an automatic system. Animal care staff provided daily visual health checks. The animal was monitored for appetite, general behavior, and stool consistency.

### Experimental procedures

**Figure 1.**
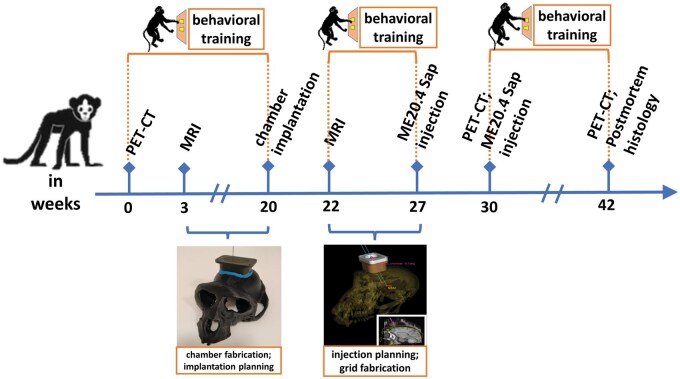
The experimental timeline, consisting of surgical planning, cognitive training and testing, surgical procedures, post-surgical evaluation. The surgical planning consisted of the NBM identification, cranial chamber and grid fabrication, and implantation planning of the cranial chamber based on the animal's MRI and CT data (Weeks 0-20, 22-27). Cognitive training was performed prior to and after the ME20.4 Saporin injections into the NBM (weeks 0-20, 22-27, and 30-42). Surgical procedures included cranial chamber implantation (week 20) and 2 injection sessions of the ME20.4 Saporin (weeks 27 and 30). The post-surgical PET-CT was performed in both week 30 and week 42 followed by sacrificing the animal and histological evaluation.

The experimental timeline, consisting of surgical planning, cognitive training and testing, surgical procedures, and post-surgical evaluation is depicted in Figure 1. First, the animal was trained with a touchscreen-based cognitive paradigm. We localized the NBM and designed the implantation of a cranial chamber based on the animal's MRI and CT data. Second, the MRI-compatible cranial chamber was implanted onto the animal's skull and the injection of ME20.4 Saporin was performed based on a second MRI. Finally, we verified the impact of the injections with both in vivo (cognitive testing and functional PET-CT imaging) and ex vivo (histology) procedures.

### Neuroimaging: PET-CT and MRI

The first PET-CT was performed as a baseline assessment of the brain cholinergic system before any of the interventions were executed. The second and third scans were performed to evaluate the effects of each of the cholinergic toxin injections into the NBM. The second scan was done 3 weeks after the first injection and surgical recovery to evaluate the immediate effect of the cholinergic toxin on the brain. The third scan was done 12 weeks after the second injection to allow cognitive assessment post-injection. For all scans, a selective presynaptic cholinergic radiotracer, [^18^F]-fluoro-ethoxy-benzo-vesamicol ([^18^F]-FEOBV), binding to the vesicular acetylcholine transporter, was used. The CT scan was used for attenuation correction and to reconstruct the animal’s skull, in order to develop a personalized cranial chamber.

T1-weighted MRIs were acquired in 2 sessions. Scans from the first session were used to locate the NBM and to plan subsequent procedures. The second scan verified the location and orientation of the implanted chamber, allowing precise calculation of the angle and depth for the trajectory of stereotactic injection.

### PET-CT

PET-CT was performed using a dedicated nonhuman primate (NHP) PET-CT, the Multiscan Large Field of View Extreme Resolution Research Imager (LFER) 150 PET-CT (Mediso Ltd, Budapest, Hungary).^27^ The animal was fasted prior to the scan and was sedated with ketamine (10 mg/kg ketamine hydrochloride, Alfasan Nederland BV, Woerden, The Netherlands) combined with medetomidine hydrochloride (0.05 mg/kg, Sedastart; AST Farma B.V., Oudewater, The Netherlands), which was injected intramuscularly (IM).

After arrival in the scanner room, artificial tears were applied to the eyes to prevent dehydration. Subsequently, the epiglottis and vocal cords were anesthetized by spraying with Xylocaine 0.1% spray (Xylocaine 100 mg/mL spray; Aspen Pharma Trading Ltd, Dublin, Ireland), followed by the insertion of an endotracheal tube, using a laryngoscope. During the scan, the animal breathed freely and anesthesia was maintained with isoflurane (0.8%-1.5% isoflurane, 50% O_2_ and 50% air, total flow 1 L/min). An intravenous (IV) catheter was inserted in the left or right saphenous vein, which was connected to a 3-way IV cannula for injection of radiotracer.

The animal was positioned headfirst supine on the scanner table in an air-heated blanket to maintain the body temperature. After the scan, the animal is extubated and upon return to his cage, atipamezole hydrochloride (0.25 mg/kg, Sedastop, ASTFarma B.V., 5 mg/ml) was administered IM to antagonize medetomidine. Heart rate, oxygen saturation level (SpO_2_%), and rectal body temperature were continuously monitored during the scan.

CT images were acquired in the axial plane in 0.6 mm or 1 mm increments (135 slices at 512 × 512; 0.24 × 0.24 mm in plane resolution). Thereafter, a bolus injection of ±76.3 MBq (range 56.0—94.4 MBq) [^18^F]-FEOBV was administered with the use of the IV cannula. Subsequently, a PET scan of the brain area was performed from 30 minutes post-injection onwards for 180 minutes. PET images were reconstructed on a 256 × 256 matrix using OSEM (8 iterations, 9 subsets) with 0.3 mm isotropic voxels including random attenuation and scatter correction.

Data were analyzed in VivoQuant 4.5 (Invicro, Boston, MA, USA). Several regions of interest (ROIs) were rendered from the digital Cortical Hierarchy Atlas of the Rhesus Macaque (CHARM) and Subcortical Atlas of the Rhesus Macaque (SARM) brain atlases that had been registered to the animal’s native space (see below). These ROIs were the basal forebrain, the amygdala, the orbitofrontal cortex, the occipital cortex, the sensorimotor cortex, and the anterior cingulum. Binding of the [^18^F]-FEOBV for each region pre-, and post-toxin injections was calculated as a standardized uptake value (SUV), expressing both the average (SUV_mean_) as well as the highest value within a 1 mm^3^ spherical volume of each region (SUV_peak_). A reduction of ≥10% in cortical ROIs and ≥15% in the basal forebrain were considered as biologically relevant. These thresholds were set based on prior studies reporting the test-retest variability for [^18^F]-FEOBV tracers.^28^^,^^29^

### Magnetic resonance imaging

Before performing the MRI, the animal was fasted overnight and transported to another location in a transport cage in an acclimatized van. Upon arrival, the monkey was sedated with ketamine (10 mg/kg IM) combined with medetomidine (0.05 mg/kg IM) before being moved to the MRI room. Subsequently, the animal was positioned headfirst prone in the MRI gantry and covered with a blanket to maintain body temperature. The heart rate and SpO_2_% were continuously monitored. Several 3D T1-weighted brain scans were acquired using a standard Philips Ingenia 3 Tesla horizontal bore full body scanner with a custom-built 8-channel phased-array receive coil system (T1-weighted, 3D-FFE, TE = 6 ms, TR = 13 ms, TI = 900 ms, flip angle = 8°, 100 horizontal slices, in-plane 224 × 224 matrix, 0.6 × 0.6 × 0.6 mm isotropic voxels, and phase-encoding in the AP direction).^30^ After the scan, atipamezole (0.25 mg/kg IM) was administered to antagonize medetomidine.

### NBM identification

The NBM is a borderless structure in the basal forebrain in both hemispheres; it is commonly identified in relation to surrounding structures. The NIH Macaque Template (NMT) was linearly and non-linearly registered to the individual anatomical MRI using AFNI’s @animal_warper program along with cortical (CHARM) and subcortical atlases of the monkey brain (SARM).^31–37^ This registration provided several ROIs in the individual’s native space. The NBM was then projected onto the MRI of the animal’s brain and the CT-based reconstruction of the skull using 3D slicer, an open-source software for biomedical image processing and image-guided procedures for scientific purposes (https://www.slicer.org/). After identification of the NBM on the MRI, these images were merged in 3D with the PET-CT in Vivoquant to enable localization of the NBM on the PET in the animal’s native space.

### Cranial chamber implantation

#### Determining chamber location

An MRI-compatible CILUX cranial chamber and access grid (Crist Instrument Co., Hagerstown, MD, USA) were used to guide a 26G injection needle used for intracranial injections. The chamber had a base size of 30 mm × 20 mm. The bottom was curved to fit the curvature of the skull, while the top side of the chamber had an expanded shoulder allowing the attachment of a microdrive or cap to close the chamber.^38^ The chamber-fitted guiding grid consists of vertical holes, with a diameter of 0.5 mm spaced 1 mm apart.

**Figure 2.**
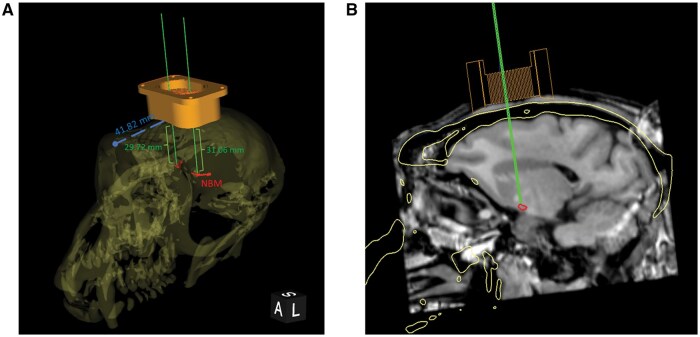
Determination of the location of the cranial chamber implantation. The location of the implantation of the cranial chamber was determined in the animal’s 3D skull-brain reconstruction based on MRI and CT data (A). The sagittal view of the planned needle trajectory visualizes the skull reconstruction (yellow line), the grid and the chamber (orange), the NBM (red), and imaginary needle (green) (B).

The location of the cranial chamber was planned using 3D slicer software. First, a 3D model of the skull was reconstructed based on the CT. A rigid transformation was then manually performed to co-register this 3D model with the brain MRI and the NBM map. Finally, a 3D model of the cranial chamber was positioned in the MRI-CT co-registered image allowing access to the NBM with insertion trajectories via the grid holes. The ear canals and the infraorbital bone were aligned in a transverse plane to allow for a similar orientation compared to fixation of the head in a stereotactic frame. In this plane, the distance between the center of the chamber and the ear canals as well as the frontal ridge of the skull were measured. Furthermore, the distance between the outer edge of the skull and the NBM was measured to determine the injection depth (Figure 2A).

#### Chamber implantation surgery

The animal was fasted overnight, presurgical analgesia, meloxicam (0.20 mg/kg IM, Metacam, Boehringer Ingelheim, Alkmaar, The Netherlands) and buprenorphine (0.04 mg/kg IM, Buprecare, AST Farma B.V.), were administered 1-hour prior to surgery. Afterwards, the animal was sedated with a combination of ketamine (10 mg/kg IM, ketamine hydrochloride, Alfasan Nederland BV) and medetomidine (0.05 mg/kg IM, Sedastart; AST Farma B.V.). No antibiotics were given, because the surgery was performed under aseptic surgical principles and the skull was not opened during this procedure.

In the operating room, the animal was intubated and was allowed to breath spontaneously throughout the surgery (0.8%-1.5% isoflurane, 20% O_2_ and 80% air, total flow 1 L/min). The animal was placed on a heated blanket to stabilize body temperature and artificial tears were applied to prevent dehydration of the eyes. The animal's head was fixed in a primate stereotactic frame (David Kopf Instruments, Tujunga, CA, USA). Heart rate, SpO_2_%, and rectal body temperature were continuously monitored during the surgery. The skin on the top of the animal's head was shaved and cleaned with Hibiscrub (chlorhexidine gluconate 40 mg/mL), followed by disinfection of the incision area with 70% ethanol. A sagittal incision of approximately 10 cm in length was made across the skin. Subcutaneous tissues and muscles were detached and retracted to expose the skull. The periosteum was scraped using a bone scraper. A cotton swab soaked in 35% hydrogen peroxide was used to degrease the surface of the skull. Subsequently, the cranial chamber was placed and fixated at the predetermined position with 4 MRI-compatible ceramic anchoring screws (Thomas Recording, Giessen, Germany). Gentamicin-loaded bone cement (Palacos R + G; Heraeus Medical GmbH, Wertheim, Germany) was use around the outside base of the chamber for implant fixation. Finally, the skin incision was closed around the chamber with VICRYL Suture 4-0 Cutting.

The animal was released from the stereotactic frame and extubated. Upon returning to his home cage, atipamezole (0.25 mg/kg IM) was administered to antagonize medetomidine. Post-surgical analgesia was provided using meloxicam (0.10 mg/kg PO) once daily in combination with buprenorphine (0.02 mg/kg IM) 3 times a day for 2 days. The chamber was cleaned using chlorhexidine gluconate (1.5 mg/mL), cetrimide (0.15 mg/mL, Hibicet Hospital Concentrate: HHC), 2% hydrogen peroxide, and physiological saline (0.9%) weekly.

#### Confirmation of chamber location

**Figure 3.**
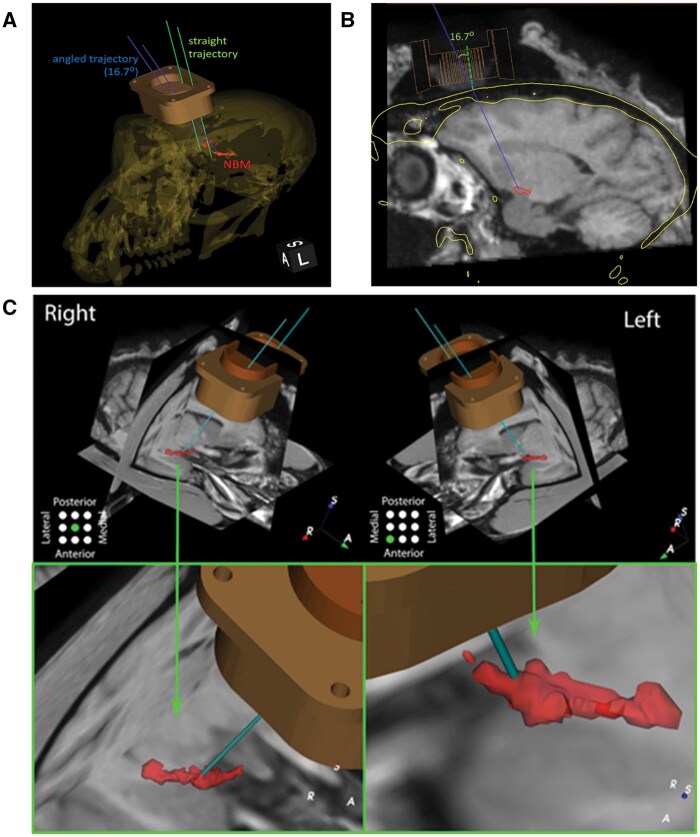
Visualization of needle entry point and depth of needle injection. Reconstruction of animal’s CT and MRI images to locate the actual cranial chamber position and the NBM indicates that straight needle trajectories (green lines) were not able to target the NBM. Hence, an angled trajectory (blue) was required (A). The angle of the injection trajectory was measured relative to the imaginary line representing a grid tunnel with a straight trajectory. This angle was used to create a custom grid with angled tunnels (B). The predicted location of the needle tip at the NBM (red) based on the planned needle entry and depth of needle insertion (C).

To verify the chamber location and determine the entry point, angle, and depth of the injection, a second MRI was performed. The same procedure was used as described above with the addition of 2 steps. First, the cranial chamber was filled with normal saline solution, which would be visible as a high-intensity signal in a T1-weighted MRI. Second, with a grid inside the chamber, 2 cannulas made of polyether ether-ketone (PEEK) were inserted into 2 of the grid holes. The PEEK cannulas are visible as low-intensity lines amidst the relatively high-intensity signals of the normal saline solution (see the ex vivo simulation, Figure 3A). Based on these markers, we reconstructed a trajectory from the chamber to the NBM. The MRI data obtained showed that the chamber was implanted slightly further anterior than planned. Consequently, there were no possible trajectories straight down from the chamber that would end up in the NBM. Therefore, an angled grid was designed. This new grid was 3D-printed and had 2 sets of 9 angled grid holes specifically designed to target the NBM in each brain hemisphere (Figure 3B). The distance between the NBM, the outer surface of the skull, and the top entry of the grid holes was re-calculated with the new materials. The needle entry point and the depth of the needle insertion were measured to ensure the needle was placed in the NBM (Figure 3C).

### Behavioral training: visual discrimination task

The behavioral task was performed in the animal’s cage using a wireless touchscreen training system adapted from an open-source program.^39^ The monkey was not forced to participate in the training: no body fixation, or food or water restriction were applied.

A classical delayed match-to-sample (DMS) task was originally attempted to assess cholinergic integrity as used in previous studies.^40^^,^^41^ However, the animal was not able to learn the task after 8 weeks of training, indicated by correct hit performance remained at chance level. We, therefore, adopted a simplified visual discrimination paradigm as described here.

The visual discrimination task was used to evaluate the animal’s memory capacity, both acquisition as well as retention, which has previously been shown to be related to the magnitude of cortical cholinergic activity.^42^ Acclimation to the use of the test system as well as the screen-based visual discrimination task was realized by several different training sessions, such as the familiarization sessions and goal oriented touching sessions. These steps took several months before the start of the study and were necessary for the monkey to learn the principles of the test. Both the cognitive training and testing were performed by an animal caretaker for approximately 15 minutes per session, each consisting of 30-60 task trials. There was only one training session per day.

**Figure 4.**
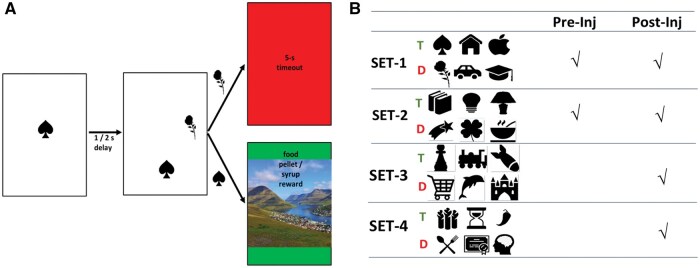
The visual discrimination task to assess cognitive function. Each trial consisted of a presentation of a target symbol, 1- or 2-s blank delay, and display of the target plus a distractor. Touching the target symbol (hit) results in a food or syrup reward, while incorrectly touching the distractor results in an additional 5-s timeout period (A). Four symbol sets were used for the test. Two sets were applied both pre- and post-injection, while 2 sets were applied only in the post-injection phase. Each set consists of 3 target symbols (T) and 3 distractor symbols (D) (B).

In the initial training phase, the animal touched a symbol presented at the center of the touchscreen or in 1 of 4 other locations (upper, lower, right, and left). A syrup/pellet reward was given after touching the symbol. After being accustomed to the touch screen, the visual discrimination task was introduced. In each trial (Figure 4), a symbol cue on a white background was presented at the center of the screen. After the animal touched the cue, the symbol cue disappeared, leaving the white blank screen for 1- or 2 seconds. Then, a pair of symbols were presented on the screen. One symbol was the target symbol, similar to the one presented as the symbol cue, while the other one was a distractor symbol. The locations of the target and the distractor were pseudo-randomized in 4 locations (upper, lower, right, and left). Touching the target symbol resulted in a landscape image and a syrup or pellet reward provided by an animal caretaker, while touching the distractor symbol resulted in a red screen and an additional 5-s timeout period. No time response limit was set. Several symbol sets were prepared, each consisting of either 2 symbols (one assigned as a distractor, and the other one as a target) for the training phase (set 1 and set 2), or 6 symbols (3 distractors, 3 targets) for the testing phase (set 3 and set 4). Reaching a hit rate of more than 70% in 3 consecutive sessions was defined as the criterium to progress to another novel symbol set. The number of sessions to reach the threshold for each symbol set, the percentage of correct responses in one session and the reaction time of each session were recorded.

In summary, 2 sets (set 1 and set 2) were done pre- and post-ME-20.4 injection to investigate the animal’s memory retention, while the other 2 sets (set 3 and set 4) were implemented only in the post-injection period in order to assess the animal’s learning capability.

### Injection of the ME20.4 Saporin

We performed a 2-stage ME20.4 Saporin injection protocol to observe a dose-effect relationship in vivo with the [^18^F]-FEOBV PET-CT. At the first injection stage, we deliberately aimed to create a partial cholinergic lesion of the bilateral NBM as occurs in the prodromal phases of AD and PD.

The first dose was chosen based on previous NBM lesioning works in common marmosets where infusing 1.4 µg ME20.4 Saporin (in a concentration of 0.20 µg/µl) into each side of the NBM produced partial NBM depletion and mild, yet measurable impairment on a visual discrimination task.^17^^,^^19^ Considering the total number of cholinergic neurons and NBM volume in rhesus macaques, which is roughly 4-5 times that of marmosets, we decided to initially start with a scaled dose of 2.5 µg (in 0.25 µg/µl ME20.4 Saporin solution).^43^^,^^44^ A second injection stage was planned if the [^18^F]-FEOBV PET-CT confirmed a partial (<90%) lesion after the first injection as was the case in this study.

At the second injection session, we administered 5 µg ME20.4 Saporin (in 0.5 µg/µl solution) into each NBM side. We hypothesized that increasing the ME20.4 Saporin dose to 7.5 µg in total, or equal to 5 times the dose in previous studies, would further deplete cholinergic neurons in the NBM and its cortical cholinergic projections. A 3-week injection interval was determined to allow postoperative recovery period, especially from acute microglial activation caused by immunotoxin and needle penetration which may confound the PET-CT measurement.^45^^,^^46^

Each injection was performed using a microliter Hamilton syringe (100 µl, Hamilton, cat. no: 1710). The syringe was moved by an electronic microdrive system adjusted to the angle of the grid (Alpha Omega). A sterile 25-gauge standard blunt needle was used (length 120 mm). The ME20.4 Saporin stock solution (Advanced Targeting Systems, Carlsbad, CA, USA) was diluted in PBS to a concentration of 0.25 µg/µl (first injection session) or 0.5 µg/µl (second injection session) and stored on ice until use.

**Figure 5.**
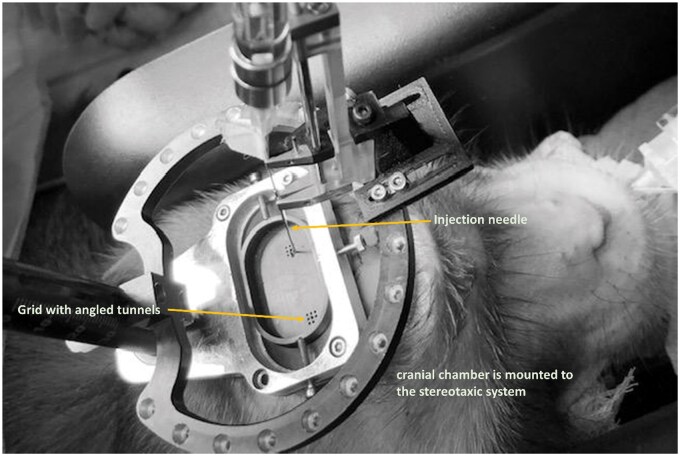
Overview of the grid, microdrive and needle in place for injection in the nucleus basalis of Meynert (NBM). The needle is gripped by the needle holder of the microdrive and is inserted through one of the predetermined angled grid holes.

The animal was fasted overnight and after sedation the head was positioned in the stereotactic frame. The microdrive system was then anchored to the cranial chamber. A small amount of methylene blue (∼5 µl) was applied to mark the injection needle entry point on the skull using a microliter syringe inserted through the predetermined grid holes. The static point of the microdrive fixing the needle was marked and the distance between the tip of the needle and the marking point was measured. After the measurement, the grid and the microdrive were disassembled from the cranial chamber and a borehole with a diameter of approximately 2.5 mm was made using a manual twist drill (Thomas Recording). A sterile 25-gauge standard sharp needle was used to puncture the dura. Then the syringe was loaded with 10 µl ME20.4 Saporin solution, mounted on the microdrive system, and guided through the grid hole which was slowly lowered towards the NBM (∼15 minutes, 31.3 mm depth for left NBM, 30.4 mm for right NBM). Once the estimated NBM location was reached, the 10 µl of ME20.4 Saporin (first stage: 2.5 µg/site; second stage 5.0 µg/site) was injected over a 5-minute period into the NBM. The needle was retracted 10 minutes after completion of the injection to allow drug penetration into the surrounding tissue. Exactly the same procedure was repeated for the contralateral NBM and also for the second injection session (Figure 5).

After completion of the second injection, the cranial chamber was removed from the skull together with the bone cement and anchoring bone screws. The skull was cleaned with hibicet, 2% hydrogen peroxide, rinsed with physiological saline, and the skin was closed with sutures. Upon return to the home cage, atipamezole (0.25 mg/kg IM) was administered to antagonize medetomidine.

### Histological examination

The animal was killed for histological examination 3 months after the last ME20.4 Saporin injection. First, the animal was anesthetized with a combination of ketamine (10 mg/kg IM) and medetomidine (0.05 mg/kg IM). Subsequently, pentobarbital (60 mg/kg) was injected IV. Necropsy was performed to isolate the brain tissue. Afterwards, the brain was separated into 2 hemispheres and fixed in 10% neutral buffered formalin solution for 48 h and cryoprotected in 30% *w*/*v* sucrose in PBS. The cerebrum was dissected in 12 different coronal blocks, cut at the anterior-posterior axis at +10, +8, +5, +1, −3, −6, −8, −11, −14, −18, and −22 mm from the bregma. The brain blocks were paraffinized and multiple consecutive 4-µm sections were sliced for immunohistochemical staining. A NBM section of an age-matched macaque from the BPRC tissue bank (https://www.bprc.nl/en/biobank) was included as a negative control.

Brain blocks containing the basal forebrain, the anterior commissure, and the amygdala were deparaffinized. Immunohistochemical staining with a primary antibody against the p75-NGF-receptor-protein, the ME20.4 monoclonal antibody, was performed to specifically identify the basal forebrain cholinergic neurons (Abcam, Cambridge, UK; catalog no. ab3125, diluted 1:100). Additionally, to observe reactive gliotic processes induced by needle penetrations, immunohistochemical staining against GFAP (Abcam, catalog no. ab68428, diluted 1:100) was performed. The staining procedure for each primary antibody was applied to every 15th brain slice, resulting in a total of 5 to 7 slices per tissue block.

First, slides were steamed for 1 h, in combination with IHC-Tek epitope retrieval solution (IHC World, LLC. Ellicott City, MD, USA) for antigen retrieval. The EnVision staining kit (G|2 double-stain system, rabbit/mouse, DAB+/permanent RED code K5361; Agilent Technologies, Glostrup, Denmark) was used. After immunohistochemical staining, the sections were dehydrated and mounted with TissueTek coverslipping film (Sakura Finetek Europe B.V., The Netherlands). Hematoxylin-eosin (HE) staining was used for general morphology. Cells positively stained with the ME20.4 antibody were counted.

## RESULTS

The animal recovered smoothly after all experimental procedures. In addition, weekly cleaning of the chamber showed no signs of infection during the entire experimental period.

### [^18^F]-FEOBV PET results

The [^18^F]-FEOBV uptake values in several brain regions-of-interest at baseline, 3 weeks post-injection 1, and 12 weeks post-injection are summarized in Table 1. The values are expressed by both the average (SUV_mean_) as well as by the highest (SUV_peak_) uptake value within regions-of interest.

**Table 1.** Summary of the findings of [^18^F]-FEOBV uptake of several brain regions-of-interest pre-injection (Pre), post-injection 1 (Post1), and post-injection 2 (Post2).

Brain area	SUVmean						SUVpeak					
	Pre	Post1	Post2	% Changes Post1-Pre	% Changes Post2-Pre	% Changes Post2-Post1	Pre	Post1	Post2	% Changes Post1-Pre	% Changes Post2-Pre	% Changes Post2-Post1
Anterior cingulum L	3,14	2,82	2,40	**−10.42 (decreased)**	**−23.72 (decreased)**	**−14.85 (decreased)**	9,26	8,85	9,86	**−**4.43 (decreased)	6.51 (increased)	11.46 (increased)
Anterior cingulum R	2,91	2,27	2,15	**−21.93 (decreased)**	**−26.14 (decreased)**	**−**5.39 (decreased)	11,17	9,40	7,49	**−15.86 (decreased)**	**−32.93 (decreased)**	**−20.28 (decreased)**
Amygdala L	3,48	2,47	2,71	**−29.09 (decreased)**	**−22.16 (decreased)**	9.77 (increased)	6,93	8,41	7,12	21.31 (increased)	2.71 (increased)	**−15.33 (decreased)**
Amygdala R	3,59	2,72	2,71	**−24.32 (decreased)**	**−24.47 (decreased)**	**−**0.20 (decreased)	6,81	7,16	8,48	5.19 (increased)	24.67 (increased)	18.52 (increased)
Hippocampus L	3,60	2,64	2,46	**−26.62 (decreased)**	**−31.48 (decreased)**	**−**6.62 (decreased)	9,85	8,10	6,55	**−17.81 (decreased)**	**−33.5 (decreased)**	**−19.09 (decreased)**
Hippocampus R	3,65	2,89	2,23	**−20.82 (decreased)**	**−38.88 (decreased)**	**−22.80 (decreased)**	9,20	9,23	6,97	0.33 (increased)	**−**24.25 (decreased)	**−24.50 (decreased)**
Occipital L	2,15	1,87	1,99	**−12.9 (decreased)**	−7.4 (decreased)	6.31 (increased)	6,73	7,43	6,81	10.48 (increased)	1.29 (increased)	**−**8.32 (decreased)
Occipital R	2,10	1,87	1,96	**−11.08 (decreased)**	−6.48 (decreased)	5.18 (increased)	6,89	8,20	7,05	19.06 (increased)	2.31 (increased)	**−14.07 (decreased)**
Orbitofrontal L	2,96	2,33	2,28	**−21.4 (decreased)**	**−22.9 (decreased)**	**−**1.90 (decreased)	11,28	7,41	6,25	**−34.26 (decreased)**	**−44.55 (decreased)**	**−15.66 (decreased)**
Orbitofrontal R	2,73	2,27	2,33	**−16.84 (decreased)**	**−14.77 (decreased)**	2.49 (increased)	8,28	6,88	7,37	**−16.96 (decreased)**	**−11.08 (decreased)**	7.08 (increased)
Sensorymotor L	2,55	2,20	2,67	**−13.69 (decreased)**	4.44 (increased)	21.01 (increased)	9,50	13,08	13,36	37.75 (increased)	40.69 (increased)	2.13 (increased)
Sensorymotor R	2,57	2,25	2,55	**−12.42 (decreased)**	−0.8 (decreased)	13.27 (increased)	13,08	12,52	13,44	**−**4.33 (decreased)	2.73 (increased)	7.38 (increased)
Basal forebrain	4,61	2,87	2,7	**−37.74 (decreased)**	**−41.43 (decreased)**	**−**5.92 (decreased)	11,11	5,51	5,64	**−50.41 (decreased)**	**−49.23 (decreased)**	2.36 (increased)

Abbreviations: SUV_mean_, the averaged standardized uptake value; SUV_peak_, the maximum standardized uptake value.

Bold values represent biologically relevant reductions, defined as ≥10% in cortical regions and ≥15% in the basal forebrain (see Discussion).

SUV_mean_ reductions were observed after the first ME20-4 Saporin injection in the basal forebrain regions and in all cortical regions-of-interest which were known to receive cholinergic terminals from the NBM. These regions include the anterior cingulum, the orbitofrontal cortex, the sensorimotor cortex, the hippocampus, the amygdala, and the occipital cortex. The reduction was ranged from 10.4% to 37.7% relative to the baseline SUV_mean_. The second injection further reduced the SUV_mean_ in the basal forebrain region, anterior cingulum (left and right hemisphere), hippocampus (left and right), the orbitofrontal cortex (left), and the amygdala (right) within the range of 0.2% to 22.8% relative to the SUV_mean_ post-first-injection. The SUV_mean_ increased at 12-week post-injection 2 for the occipital cortex (left and right), the sensorimotor cortex (left and right), the amygdala (left), and the orbitofrontal cortex (right).

Decreased SUV_peak_ was similarly observed post-injection 1 in the basal forebrain region, the anterior cingulum (left and right hemisphere), hippocampus (left), orbitofrontal cortex (left and right), and the sensorimotor cortex (right). The second ME20.4 Saporin injection further lowered the SUV_peak_ in the anterior cingulum (right hemisphere), the hippocampus (left), and the orbitofrontal cortex (left) with a range from 15.7% to 20.3%. In contrast, SUV_peak_ increased post-ME20.4 Saporin injection in the right amygdala and the left sensorimotor cortex.

### Cognitive evaluations

#### Set-1 and set-2: effect of ME20.4 Saporin in long-term memory

Due to the habituation period with the first set of symbols, before the start of the actual task, the animal immediately reached a minimum target hit percentage of at least 70%. Thereafter, the animal was able to maintain the 70% hit threshold over all the pre-injection sessions and the post-injection sessions.

For the second set completely new symbols were used. The animal was able to reach the 70% hit threshold in the second session, which sustained for the 3 consecutive sessions. Decreased performances, with this second set, were observed within the last 4 sessions before the ME20.4 Saporin injection. Post-injection, the animal re-gained the minimum 70% hit starting from the second test session, which remained stable afterward.

No significant differences were observed between the hit rate percentages pre- and post- ME20.4 Saporin injection, while the animal’s response time post-injection was significantly shorter compared to pre-injection. Similar results were observed for the 2nd symbol set. No effect on the animal’s memory retention was found.

#### Set-3 and set-4: effects of ME20.4 Saporin injection on learning

All symbols in set-3 and set-4 were newly introduced in the post-injection phase. The animal reached the stable minimum learning threshold (70% for 3 consecutive sessions) after 5 and 2 sessions, respectively. Although one of the newly introduced symbol sets showed a slight decline in reaching the 70% criterium, this did not affect the animal’s overall learning capability.

### Histological results

#### Effect on cholinergic cells

**Figure 6.**
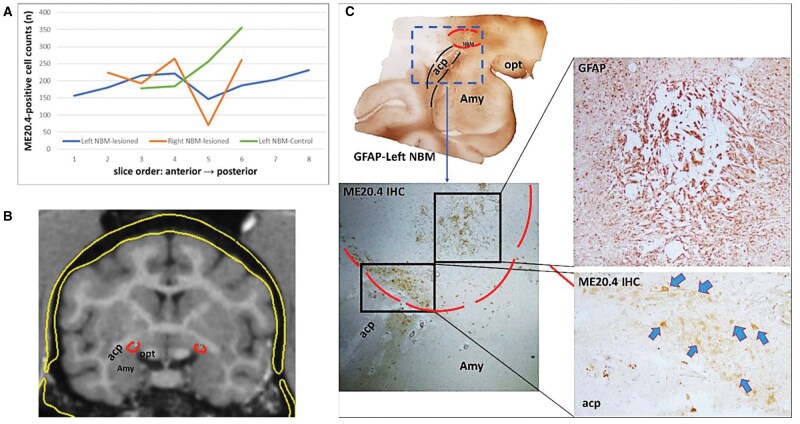
Postmortem verification of the ME20.4 Saporin injection to the NBM. The total number of ME20.4-positive cells in a series of slides, representing the anterior part of the NBM, marked by the presence of the amygdala and the anterior commissure. The brain slices counted here were ±180 µm apart, arranged from anterior to posterior (A). The coronal MRI section shows the NBM (in red) and the injection plan with the needle tip in the NBM (in turquoise) (B). Immunohistochemical assessments confirmed the injection location as planned and showed the effects of the cholinergic immunotoxin injection which induces gliotic processes surrounding the needle track (GFAP staining) and a decreased number of cholinergic cells (arrows) shown by ME20.4 staining (C). Abbreviations: Amy = amygdala; acp = posterior anterior commissure; NBM = nucleus basalis of Meynert.

A local decrease of ME20.4-positive cells in one coronal slice (slice 5) compared to subsequent slices of the same individual and to slices from the negative control animal were observed (Figure 6A-C). Compared with the negative control, a 42% cholinergic-cell reduction on the left and a 76% reduction on the right NBM were observed in slice 5, which corresponds to the intermediate part of the NBM. When the 4 coronal slices that were matched with the control slices (slices 3-6) were pooled, the cholinergic-positive loss was modified to 21%-22%.

Histological assessments verified the concordance of the needle tip location between the MRI-based trajectory and the actual injection (Figure 6B and C). The injection trajectory was traced postmortem by glial processes induced by the needle penetration.

## DISCUSSION

This study shows the feasibility of targeting NBM for intracranial injections using an extracranial implantation chamber and guidance grid, combined with pre-surgical PET-CT and MR imaging. This approach allowed personalized and reproducible stereotactic delivery of a cholinergic toxin into the NBM.^16^^,^^24–26^

The lesioning effect of the repeated ME20.4 Saporin injection was locally identified in the NBM by [^18^F]-FEOBV PET as well as postmortem histology. No clear behavioral effects were observed in the present behavioral task, a simplified form of the classical DMS paradigm, which ordinarily relies on intact cholinergic function.^40^^,^^42^ The non-measurable cognitive effect might be due to the partial state of the NBM cholinergic lesion and due to cholinergic compensatory upregulation of the remaining cholinergic neurons, which might have been induced by acute depletion of cholinergic neurons in the NBM.

Additionally, the unaffected cognitive measure may be attributable to the modest mnemonic load of the visual discrimination task implemented in this study. Compared with the classical DMS paradigm, our task imposed a simpler rule where symbols used as targets and distractors were pre-assigned and remained distinct throughout testing. This rule eliminated the need for the animal to update a match/non-match rule applied in the classical DMS paradigm. Additionally, the short delay interval (1-2 s) applied in this task could contribute to the modest mnemonic demand. In previous reports, animals with partial cholinergic depletion were still able to maintain their performance in a low-demanding mnemonic task.^47^ Measurable disruption on object discrimination task, on the other hand, was reported in a near-complete cholinergic ablation applied in marmosets.^48^ Increasing the mnemonic load by lengthening the delay period for each trial and or enhancing the cholinergic lesioning may therefore cause observable worsening in the current task.

In this study, the second MRI showed that the location of the chamber was suboptimal. This implicates that in vivo fiducial markers for location determination, such as the animal’s orbital ridge and ear canals, were insufficient for proper target localization. Furthermore, scalp muscles can slightly move the cranial chamber during the fixation process, as occurred in this experiment. The inaccuracy in chamber implantations can potentially be prevented by adding non-bony fiducial markers, in addition to the in vivo bony landmarks, to meticulously calculate the location of the chamber implantation.^22^ For example, earlier studies conducted MRI with the animals secured in an MRI-compatible stereotactic frame, with 2 ear bars containing vitamin E and a pointer-mounted micromanipulator to locate the upper lateral incisors as fiducial markers.^49^^,^^50^ Alternatively, a cranial chamber can be implanted using approximate bony landmarks and a deep brain target, provided a custom angled grid can be fabricated in-house. This way, only a single MRI session is needed to determine the custom grid angle and needle length required to reach the deep brain target. In addition to offline planning based on CT/MR,^16^^,^^24–26^^,^^49^ frameless neuronavigation solutions now also exist that allow registration of the physical animal in the surgical suite to its digital counterpart in the planning software.^20^^,^^51^

Some deep brain nuclei can be visualized with specific MRI sequences but this is not yet possible for the NBM due to its non-encapsulated, borderless structural features. Therefore, we indirectly delineated the NBM by coregistering it with the recently published digital Subcortical Atlas of the Rhesus Macaque (SARM). This atlas contains multi-level cortical parcellations (6 levels, from broad to more refined subdivisions) and offers opportunities to localize small structures such as the NBM (∼285 mm^3^).^34^ Its use for fMRI data analyses in an NHP brain template has been validated, allowing more flexible and precise ROI-based analyses. In our case, the digital atlas was back registered to animal’s native space. A previous study showed significantly improved spatial accuracy when a digital atlas was warped to an animal's native space rather than using standardized stereotactic coordinates directly from a common template brain.^52^

The limited spatial effects of the ME20.4 Saporin injection may be caused by insufficient toxin doses or limited toxin diffusion to surrounding brain tissues. Previous studies in a smaller NHP (common marmoset, *Callithrix jacchus*), applied significantly lower doses (3.5 µl; concentration 0.1 µg/µl) compared to the doses used in our study (maximum dose 10 µl in 0.5 µg/µl). However, these studies showed a significant lesioning of NBM neurons and depletion of cortical acetylcholinesterase.^17–19^ Injecting the toxin at multiple sites, as performed in those studies, could possibly improve the distribution of the toxin around the NBM areas. However, one should be aware that repetitive injections may increase the risk of intracerebral hemorrhage and associated side effects.

The [^18^F]-FEOBV radiotracer binds to the vesicular acetylcholine transporter (VAChT), which is specifically localized in cholinergic neurons. Its use in PET-CTs allows the visualization of the cholinergic integrity level.^29^ Prior studies reported the test-retest variability for [^18^F]-FEOBV binding between 5-10% in cortical regions and up to 17% in the basal ganglia.^28^^,^^29^ Therefore, any SUVR change above 10% in cortical ROIs and 17% in the basal forebrain might be considered as a relevant change. In this study, the effect of lesioning the NBM was more pronounced for the proximal NBM-projected regions (10%-40% reduced uptake), such as the basal forebrain, anterior cingulum, amygdala, and orbitofrontal cortex, compared to the more distant cortical projection sites such as the sensorimotor and the occipital cortices (∼10% reduction). The reduction magnitudes observed in this study closely mirror clinical data where 20%-38% cholinergic losses were observed in non-demented PD and AD. However, a different topographical pattern of cholinergic loss was observed in PD in which posterior cholinergic loss was more pronounced. The topographical differences might result from post-lesioning restorative mechanisms, which might be more effective in the distant regions. An alternative explanation is that the variable cortical effects are related to the patchy lesioning of the NBM.^53^

The smaller reduction in [^18^F]-FEOBV signals after the second toxin injection compared to the first and the lack of behavioral impact may be due to compensatory cholinergic upregulation.^54^ This aligns with clinical evidence showing increased cholinergic activity in the prodromal stages of AD and PD and in newly diagnosed PD patients without cognitive impairment.^55–58^ This compensation may result from structural changes, increased cholinergic synaptic density, and increased acetylcholine production in response to acute lesioning. The presence of unaffected NBM cholinergic neurons likely allows for compensation. To confirm this, further studies with a more extensively lesioned NBM and long-term follow-up are needed. Previous rodent studies indicate that at least 40% to 60% of basal forebrain cholinergic neurons have to be lesioned, in order to observe behavioral effects.^59^ This also seems to explain the lack of significant effects from stimulating NBM neurons in dementia patients, as the number of remaining cholinergic neurons apparently is not enough to compensate for the depletion.

In conclusion, MRI-guided stereotactic injection of Saporin into the nucleus basalis of Meynert (NBM) of a Rhesus monkey causes selective depletion of cholinergic neurons. ME20.4 Saporin-mediated selective destruction of NBM neurons offers a model to mimic neurodegenerative diseases and to study neuromodulatory approaches of the NBM. The precise dose-effect relationship and the optimal cognitive endpoints in Rhesus monkeys to evaluate the effects of interventions need to be established.
